# Progressing Backwards

**Published:** 1911-06-15

**Authors:** H. T. Webster


### PROGRESSING BACKWARDS.

BY H. T. WEBSTER, M.D.

In olden time before men became so wise, candy was not only a tooth-some tidbit, but it was not so very objectionable; for it was made from sugar, and while too much sugar may be objectionable, in moderate quantities it can hardly be considered poisonous. But in late years it has been found that a kind of glucose can be prepared by treating corn, corn-cobs, sawdust, etc., with sulphuric acid, and a sugar is thus prepared which costs about one-fourth as much as good cane sugar.

Of course, like other folks, the candy-makers are on the make, not only on candy but on profits, and now it is asserted that a large bulk of the candy that is offered for sale is made up of such cheap gulcose. Sulphuric acid may be good for some purposes, but it would seem hardly a good thing as a common diet for children and other lovers of candy.

An expose on this subject, which appears in the February number of *Hampton's Magazine* is interesting reading and especially to be recommended to candy lovers and parents. The writer has been called several times within recent years to treat sick children whose ailments were ascribed by their parents to an overgorging with candy. No wonder, if half of what is told about the preparation of the kind of stuff dispensed by candy-makers is true!

Such material causes sticking sensations in the throat and a constrictive pain in the stomach for twenty-four hours in anyone who is not possessed of a cast-iron digestion. And after all is over, not quite all is over, for the foundation of some serious organic malady may be laid, especially if the candy habit is persisted in.

In spite of the pure food law there is no doubt that many of the tinned goods in the market, indeed the bulk of them, contain either sweetening from such source, or else preservatives which are most objectionable, such as formaldehyde, boric acid, etc., the common use of which is certainly injurious to health.

It seems as though the only protection the public has nowadays against poisonous food is to put up its own canned goods, raise its own meat, and avoid candy. Large corporations are making their millions putting up rotten and poisonous stuff disguised with chemicals for the consumption of the people, and steadily poisoning them to death. Of course the poisoning may be insidious, but nevertheless it tends to feeble vitality, organic disease of the intestinal tract and destruction of the red blood corpuscles.

H. P. Cassidy, food inspector of Philadelphia, found enough aniline in a dish of strawberry ice-cream to color a bright crimson spot on a piece of cloth, and in an ordinary box of marshmallows enough furniture glue to paste two pieces of wood together so firmly as to support the weight of a flatiron.

He found also that it was common practice among the retail butchers to treat scraps of questionable meat with "Jerusalem," a preparation of sulphites, which imparted a bright-red color to the mess and disguised the putrescency, thus making the stuff attractive as hamburger steak.

The same course of investigation demonstrated many other dangerous combinations offered for food consumption. Among other things it was found that confectioners were selling cakes frosted with starch and sweetened soap bark; Easter eggs and almonds glossed over with common furniture shellac dissolved with wood alcohol, which causes blindness; many cheap candies—"Foxy Grandpas,"bloomer girls, aerials, and a variety of marshmallows—all containing furniture glue bleached with sulphites; cheap chocolate drops that were simply lumps of tallow and grease smeared over with a mixture of iron rust, sugar and a little chocolate; a popular candy with a banana flavor, the flavor being nothing else than the commercial lacquer that is mixed with bronzing and used for painting steam radiators.

Think of it!

It is well also to avoid corn-flavored syrups put up for consumption on hot cakes, as these are probably made up largely of the cheap glucose already referred to. They may please the taste and tickle the palate, but there may finally be death in them. A company in California, recently organized, is preparing to furnish to the grocery trade a class of syrups made from prunes and raisins, which will represent a sweetening that is pure and wholesome. They will be made from ripe prunes and raisins, and cannot be objectionable. The writer, having used samples of these, can testify to their palatability, and to the efficacy of the prune syrup, especially, as a gentle laxative, thus especially recommending it to those troubled with habitual constipation. —*Eclectic Medical Journal.*

